# Supplementary material for: Improved use of a public good selects for the evolution of undifferentiated multicellularity
Source: eLife. 2013 Apr 2;2:e00367. doi: 10.7554/eLife.00367 (PMC3614033; doi:10.7554/eLife.00367)
Supplement: Supplementary file 2. — Putative causal mutation pathway summary. DOI: http://dx.doi.org/10.7554/eLife.00367.025 [file elife00367s002.doc]

### Supplementary File 2: Putative causal mutation pathway summary

| Strain name | *ACE2* mutation | *CTS1*, *DSE1,* or *DSE2* tenfold reduction in expression | *UBR1* mutation | *RGT1* pathway mutation | Mediator mutation | *IRA1/2* mutation | *HXT* increases |
| --- | --- | --- | --- | --- | --- | --- | --- |
| EvoClone1 | nonsense | All 3 | nonsense | *RGT1* nonsense | *SIN4* missense | *IRA1* missense | 1,2,3,4 |
| EvoClone2 | nonsense | All 3 | missense | *MTH1* nonsense | *CSE2* nonsense | *IRA1* missense | 4 |
| EvoClone3 |  | All 3 |  |  |  | *IRA2* missense | 4 |
| EvoClone4 | nonsense | All 3 | nonsense | *RGT1* nonsense | *SIN4* missense |  | 1,3,4,9,11 |
| EvoClone5 | nonsense | All 3 |  | *SNF3* missense |  |  | 1,2,3,4,6,7 |
| EvoClone6 | nonsense | All 3 |  | *MTH1* nonsense |  |  | 2,4 |
| EvoClone7A | nonsense | All 3 |  |  |  |  | 2,3,4 |
| EvoClone7B |  |  |  | *SNF3* missense |  | *IRA2* nonsense | 2,3,4 |
| EvoClone7C |  | All 3 |  |  |  | *IRA2* nonsense | 1,2,3,4,6 |
| EvoClone8 | promoter | *CTS1* only | nonsense | *SNF3* missense |  |  | 2,3,4 |
| EvoClone9 |  | All 3 | nonsense |  | *MED1* nonsense |  | 2 |
| EvoClone10 | missense |  | missense | *RGT1* missense | *NUT1* nonsense |  |  |
