# Supplementary material for: Improved use of a public good selects for the evolution of undifferentiated multicellularity
Source: eLife. 2013 Apr 2;2:e00367. doi: 10.7554/eLife.00367 (PMC3614033; doi:10.7554/eLife.00367)
Supplement: Supplementary file 3. — Strain list. DOI: http://dx.doi.org/10.7554/eLife.00367.026 [file elife00367s003.doc]

Supplementary File 3 – Strain list

| Systematic name | Description in text | Constitutive color marker | Genotype |
| --- | --- | --- | --- |
| yJHK085 | (Note 1) | None | *his3-11,15*; *ura3∆0* |
| yJHK089 | (Note 3) | None | *his3-11,15* |
| yJHK110 | Ancestor derivative (note 3) | *his3∆*::*P_ACT1_*-yCerulean-*tADH1*-*His3MX6* |  |
| yJHK111 | Ancestor derivative (note 3) | *his3∆*::*P_ACT1_*-ymCitrine-*tADH1*-*His3MX6* |  |
| yJHK112 | Ancestor derivative (note 3) | *his3∆*::*P_ACT1_*-ymCherry-*tADH1*-*His3MX6* |  |
| yJHK152 | *AMN1-RM11*, CFP labeled (Fig 4); EngClumpy competed vs. wild-type lab (Table 1) | *his3∆*::*P_ACT1_*-yCerulean-*tADH1*-*His3MX6* | *AMN1-RM11* |
| yJHK153 | *AMN1-RM11*, YFP labeled (Fig 4); EngClumpy competed vs. EvoClone2 (Table 1) | *his3∆*::*P_ACT1_*-ymCitrine-*tADH1*-*His3MX6* | *AMN1-RM11* |
| yJHK222 | *SUC2*, no import (Fig 2); wt (Table 4) | *his3∆*::*P_ACT1_*-ymCitrine-*tADH1*-*His3MX6* | *mal11/12*∆::*hphMX4* |
| yJHK288 | wt-*suc2-1cyt* (Table 4) | *his3∆*::*P_ACT1_*-yCerulean-*tADH1*-*His3MX6* | *suc2-1cyt*  *mal11/12*∆::*hphMX4* |
| yJHK312 | Induced invertase (Fig 2); EngHiInvertase (Table 1) | *ura3∆*::*P_ACT1_*-ymCitrine-*tADH1-URA3* | *P_SUC2_*∆::*kanMX6*-*P_GAL1_*-*SUC2*  *gal1/10*∆::*LEU2*  *P_GAL3_*∆::*His3MX6*-*P_ACT1_*-*GAL3*  *mal11/12*∆::*hphMX4* |
| yJHK369 | wild-type competed against EngHiInvertase (Table 1) | *ura3∆*::*P_ACT1_*-yCerulean-*tADH1-URA3* | *gal1/10*∆::*LEU2*  *P_GAL3_*∆::*His3MX6*-*P_ACT1_*-*GAL3*  *mal11/12*∆::*hphMX4* |
| yJHK372 | *SUC2*, import (Fig 2); EngSucImport (Table 1) | *his3∆*::*P_ACT1_*-ymCitrine-*tADH1*-*His3MX6* | *ho*∆::*P_ACT1_*-*MAL11*-*tADH1*-*kanMX6*  *mal11/12*∆::*hphMX4* |
| yJHK373 | *suc2-1cyt*, import (Fig 2); wt-*suc2-1cyt* import (Table 4) | *his3∆*::*P_ACT1_*-ymCitrine-*tADH1*-*His3MX6* | *suc2-1cyt*  *ho*∆::*P_ACT1_*-*MAL11*-*tADH1*-*kanMX6*  *mal11/12*∆::*hphMX4* |
| yJHK374 | *suc2*∆, import (Fig 2) | *his3∆*::*P_ACT1_*-ymCitrine-*tADH1*-*His3MX6* | *suc2*∆::*natMX4*  *ho*∆::*P_ACT1_*-*MAL11*-*tADH1*-*kanMX6*  *mal11/12*∆::*hphMX4* |
| yJHK407 | wild-type competed against EngSucImport (Table 1) | *his3∆*::*P_ACT1_*-yCerulean-*tADH1*-*His3MX6* | *ho*∆::*kanMX6*  *mal11/12*∆::*hphMX4* |
| yJHK459 | DNA-sequenced common ancestor of all evolved strains (note 4). |  | *ura3∆0* |
| yJHK469 | (Note 4) |  | *pol3-L523D*  *ura3∆0* |
| yJHK470 | (Note 4) |  | *pol3-L523D*  *ura3∆0* |
| yJHK471 | Starting strain for EvoPopulation3 (note 4) | *ura3∆*::*P_ACT1_*-ymCitrine-*tADH1-URA3* | *pol3-L523D* |
| yJHK472 | Starting strain for EvoPopulation1 and EvoPopulation4 (note 4) | *ura3∆*::*P_ACT1_*-ymCitrine-*tADH1-URA3* | *pol3-L523D* |
| yJHK473 | Starting strain for EvoPopulation5 (note 4) | *ura3∆*::*P_ACT1_*-ymCitrine-*tADH1-URA3* | *pol3-L523D* |
| yJHK475 | Starting strain for EvoPopulation6 (note 4) | *ura3∆*::*P_ACT1_*-ymCitrine-*tADH1-URA3* | *pol3-L523D* |
| yJHK476 | Starting strain for EvoPopulation7 (note 4) | *ura3∆*::*P_ACT1_*-ymCitrine-*tADH1-URA3* | *pol3-L523D* |
| yJHK481 | Starting strain for EvoPopulation2 and EvoPopulation8 (note 4) | *ura3∆*::*P_ACT1_*-ymCitrine-*tADH1-URA3* | *pol3-L523D*  *mal11/12*∆::*hphMX4* |
| yJHK482 | Starting strain for EvoPopulation9 (note 4) | *ura3∆*::*P_ACT1_*-ymCitrine-*tADH1-URA3* | *pol3-L523D*  *mal11/12*∆::*hphMX4* |
| yJHK483 | Starting strain for EvoPopulation10 (note 4) | *ura3∆*::*P_ACT1_*-ymCitrine-*tADH1-URA3* | *pol3-L523D*  *mal11/12*∆::*hphMX4* |
| yJHK518 | *ace2*∆ (Fig 8) | *his3∆*::*P_ACT1_*-yCerulean-*tADH1*-*His3MX6* | *ace2*∆:*ble* |
| yJHK519 | *MAT****α**** *ancestor for bulk segregant analysis (note 5) | *his3∆*::*P_ACT1_*-yCerulean-*tADH1*-*His3MX6* | *trp1∆::kanMX4*  *ho∆::P_MFA_1-*ymCherry*-hphMX4*  *P_URA3_∆::NatMX4-P_STE2_* |
| yJHK529 | EvoClone1 | *ura3∆*::*P_ACT1_*-ymCitrine-*tADH1-URA3* | *pol3-L523D* |
| yJHK530 | EvoClone2 | *ura3∆*::*P_ACT1_*-ymCitrine-*tADH1-URA3* | *pol3-L523D*  *mal11/12*∆::*hphMX4* |
| yJHK531 | EvoClone3 | *ura3∆*::*P_ACT1_*-ymCitrine-*tADH1-URA3* | *pol3-L523D* |
| yJHK532 | EvoClone4 | *ura3∆*::*P_ACT1_*-ymCitrine-*tADH1-URA3* | *pol3-L523D* |
| yJHK533 | EvoClone5 | *ura3∆*::*P_ACT1_*-ymCitrine-*tADH1-URA3* | *pol3-L523D* |
| yJHK534 | EvoClone6 | *ura3∆*::*P_ACT1_*-ymCitrine-*tADH1-URA3* | *pol3-L523D* |
| yJHK535 | EvoClone7A | *ura3∆*::*P_ACT1_*-ymCitrine-*tADH1-URA3* | *pol3-L523D* |
| yJHK536 | EvoClone7B | *ura3∆*::*P_ACT1_*-ymCitrine-*tADH1-URA3* | *pol3-L523D* |
| yJHK537 | EvoClone7C | *ura3∆*::*P_ACT1_*-ymCitrine-*tADH1-URA3* | *pol3-L523D* |
| yJHK538 | EvoClone8 | *ura3∆*::*P_ACT1_*-ymCitrine-*tADH1-URA3* | *pol3-L523D*  *mal11/12*∆::*hphMX4* |
| yJHK539 | EvoClone9 | *ura3∆*::*P_ACT1_*-ymCitrine-*tADH1-URA3* | *pol3-L523D*  *mal11/12*∆::*hphMX4* |
| yJHK540 | EvoClone10 | *ura3∆*::*P_ACT1_*-ymCitrine-*tADH1-URA3* | *pol3-L523D*  *mal11/12*∆::*hphMX4* |
| yJHK588 | *gin4-W19* irc8-G57V mck1-G227Vfs249* (Fig 8, Table 1) | *his3∆*::*P_ACT1_*-yCerulean-*tADH1*-*His3MX6* | *gin4-W19**  *irc8-G57V*  *mck1-G227Vfs249*  *MYO1*-ymCitrine-*natMX4* |
| yJHK599 | EvoClone1-CFP | *ura3∆*::*P_ACT1_*-yCerulean-*tADH1-URA3* | *pol3-L523D* |
| yJHK600 | EvoClone2-CFP | *ura3∆*::*P_ACT1_*-yCerulean-*tADH1-URA3* | *pol3-L523D*  *mal11/12*∆::*hphMX4* |
| yJHK601 | EvoClone3-CFP | *ura3∆*::*P_ACT1_*-yCerulean-*tADH1-URA3* | *pol3-L523D* |
| yJHK602 | EvoClone4-CFP | *ura3∆*::*P_ACT1_*-yCerulean-*tADH1-URA3* | *pol3-L523D* |
| yJHK603 | EvoClone5-CFP | *ura3∆*::*P_ACT1_*-yCerulean-*tADH1-URA3* | *pol3-L523D* |
| yJHK604 | EvoClone6-CFP | *ura3∆*::*P_ACT1_*-yCerulean-*tADH1-URA3* | *pol3-L523D* |
| yJHK605 | EvoClone7A-CFP | *ura3∆*::*P_ACT1_*-yCerulean-*tADH1-URA3* | *pol3-L523D* |
| yJHK606 | EvoClone7B-CFP | *ura3∆*::*P_ACT1_*-yCerulean-*tADH1-URA3* | *pol3-L523D* |
| yJHK607 | EvoClone7C-CFP | *ura3∆*::*P_ACT1_*-yCerulean-*tADH1-URA3* | *pol3-L523D* |
| yJHK608 | EvoClone8-CFP | *ura3∆*::*P_ACT1_*-yCerulean-*tADH1-URA3* | *pol3-L523D*  *mal11/12*∆::*hphMX4* |
| yJHK609 | EvoClone9-CFP | *ura3∆*::*P_ACT1_*-yCerulean-*tADH1-URA3* | *pol3-L523D*  *mal11/12*∆::*hphMX4* |
| yJHK610 | EvoClone10-CFP | *ura3∆*::*P_ACT1_*-yCerulean-*tADH1-URA3* | *pol3-L523D*  *mal11/12*∆::*hphMX4* |
| yJHK630 | EvoClone1-*suc2-1cyt* | *ura3∆*::*P_ACT1_*-yCerulean-*tADH1-URA3* | *pol3-L523D*  *suc2-1cyt* |
| yJHK631 | EvoClone2-*suc2-1cyt* | *ura3∆*::*P_ACT1_*-yCerulean-*tADH1-URA3* | *pol3-L523D*  *suc2-1cyt*  *mal11/12*∆::*hphMX4* |
| yJHK632 | EvoClone3-*suc2-1cyt* | *ura3∆*::*P_ACT1_*-yCerulean-*tADH1-URA3* | *pol3-L523D*  *suc2-1cyt* |
| yJHK635 | EvoClone6-*suc2-1cyt* | *ura3∆*::*P_ACT1_*-yCerulean-*tADH1-URA3* | *pol3-L523D*  *suc2-1cyt* |
| yJHK636 | EvoClone7A-*suc2-1cyt* | *ura3∆*::*P_ACT1_*-yCerulean-*tADH1-URA3* | *pol3-L523D*  *suc2-1cyt* |
| yJHK637 | EvoClone7B-*suc2-1cyt* | *ura3∆*::*P_ACT1_*-yCerulean-*tADH1-URA3* | *pol3-L523D*  *suc2-1cyt* |
| yJHK638 | EvoClone7C-*suc2-1cyt* | *ura3∆*::*P_ACT1_*-yCerulean-*tADH1-URA3* | *pol3-L523D*  *suc2-1cyt* |
| yJHK641 | EvoClone10-*suc2-1cyt* | *ura3∆*::*P_ACT1_*-yCerulean-*tADH1-URA3* | *pol3-L523D*  *suc2-1cyt*  *mal11/12*∆::*hphMX4* |
| yJHK680 | Recreated2;  *ace2*, *cse2, ira1, mth1, ubr1* for qPCR (Fig 5 – supp 1) | *his3∆*::*P_ACT1_*-yCerulean-*tADH1*-*His3MX6* | *ace2-L323**  cse2-S35Rfs54  *ira1-S2553C*  *mth1-H154Tfs156*  *ubr1-C175Y*  *MYO1*-ymCitrine-*natMX4* |
| yJHK682 | Reverted2 | *his3∆*::*P_ACT1_*-yCerulean-*tADH1*-*His3MX6* | *pol3-L523D*  *ACE2-W303*  *CSE2-W303*  *IRA1-W303*  *MTH1-W303*  *UBR1-W303*  *mal11/12∆::hphMX4* |
| yJHK683 | Recreated9 | *his3∆*::*P_ACT1_*-yCerulean-*tADH1*-*His3MX6* | *are1--10G>T*  *gcn2-I1528L*  *gin4-W19**  *irc8-G57V*  *mcd1-S175L*  *mck1-G227Vfs249*  *med1-L337V,E489**  *ubr1-L1050Yfs1063*  *MYO1*-ymCitrine-*natMX4* |
| yJHK689 | EvoClone8-*suc2-1cyt* | *ura3∆*::*P_ACT1_*-yCerulean-*tADH1-URA3* | *pol3-L523D*  *suc2-1cyt*  *mal11/12*∆::*hphMX4* |
| yJHK695 | *FLO1*-CFP (Fig 4) | *ura3∆*::*P_ACT1_*-yCerulean-*tADH1-URA3* | *P_FLO1_*-*FLO1*::*kanMX6*-*P_TEF_*-*FLO1*  *his3-1*  *leu2∆0*  *lys2∆0* |
| yJHK696 | *FLO1*-YFP (Fig 4) | *ura3∆*::*P_ACT1_*-ymCitrine-*tADH1-URA3* | *P_FLO1_*-*FLO1*::*kanMX6*-*P_TEF_*-*FLO1*  *his3-1*  *leu2∆0*  *lys2∆0* |
| yJHK701 | EvoClone5-*suc2-1cyt* | *ura3∆*::*P_ACT1_*-yCerulean-*tADH1-URA3* | *pol3-L523D*  *suc2-1cyt* |
| yJHK721 | EvoClone4-*suc2-1cyt* | *ura3∆*::*P_ACT1_*-yCerulean-*tADH1-URA3* | *pol3-L523D*  *suc2-1cyt* |
| yJHK723 | EvoClone9-*suc2-1cyt* | *ura3∆*::*P_ACT1_*-yCerulean-*tADH1-URA3* | *pol3-L523D*  *suc2-1cyt*  *mal11/12*∆::*hphMX4* |
| yJHK725 | Reverted9 | *his3∆*::*P_ACT1_*-yCerulean-*tADH1*-*His3MX6* | *pol3-L523D*  *ARE1-W303*  *GCN2-W303*  *GIN4-W303*  *IRC8-W303*  *MCD1-W303*  *MCK1-W303*  *MED1-W303*  *UBR1-W303*  *mal11/12∆::hphMX4* |
| yJHK750 | Ancestor for RT-qPCR (Fig 8 – supp 1) | *his3∆*::*P_ACT1_*-yCerulean-*tADH1*-*His3MX6* | *MYO1*-ymCitrine-*natMX4* |
| yJHK751 | *ira1* for RT-qPCR (Fig 8 – supp 1) | *his3∆*::*P_ACT1_*-yCerulean-*tADH1*-*His3MX6* | *ira1-S2553C*  *MYO1*-ymCitrine-*natMX4* |
| yJHK752 | *ira1 mth1* for RT-qPCR (Fig 8 – supp 1) | *his3∆*::*P_ACT1_*-yCerulean-*tADH1*-*His3MX6* | *ira1-S2553C*  *mth1-H154Tfs156*  *MYO1*-ymCitrine-*natMX4* |
| yJHK753 | *ace2 ira1 mth1* for RT-qPCR (Fig 8 – supp 1) | *his3∆*::*P_ACT1_*-yCerulean-*tADH1*-*His3MX6* | *ace2-L323**  *ira1-S2553C*  *mth1-H154Tfs156*  *MYO1*-ymCitrine-*natMX4* |
| yJHK754 | *ace2 ira1 mth1 ubr1* for RT-qPCR (Fig 8 – supp 1) | *his3∆*::*P_ACT1_*-yCerulean-*tADH1*-*His3MX6* | *ace2-L323**  *ira1-S2553C*  *mth1-H154Tfs156*  *ubr1-C175Y*  *MYO1*-ymCitrine-*natMX4* |
| yJHK755 | *ubr1* for RT-qPCR (Fig 8 – supp 1) | *his3∆*::*P_ACT1_*-yCerulean-*tADH1*-*His3MX6* | *ubr1-C175Y*  *MYO1*-ymCitrine-*natMX4* |
| yJHK756 | *mth1* for RT-qPCR (Fig 8 – supp 1) | *his3∆*::*P_ACT1_*-yCerulean-*tADH1*-*His3MX6* | *mth1-H154Tfs156*  *MYO1*-ymCitrine-*natMX4* |
| yJHK757 | *ace2* for RT-qPCR (Fig 8 – supp 1) | *his3∆*::*P_ACT1_*-yCerulean-*tADH1*-*His3MX6* | *ace2-L323**  *MYO1*-ymCitrine-*natMX4* |

Strain notes:

1. All strains except for yJHK695 and yJHK696 are descendants of yJHK085, a W303 background, *MAT****a****, can1-100* strain with the S288C (corrected) allele of *BUD4* (standard W303 strains have a mutation in *BUD4* ). *BUD4* was corrected by the authors using plasmid pJHK047. All strains listed here are heterothallic.

2. yJHK695 and yJHK696 were derived from a *FLO1*-overexpressing BY4742 (S288C) background strain , which was provided by the Verstrepen lab.

3. yJHK085 is the ancestor of yJHK089, which is the ancestor of yJHK110, 111, and 112, the fluorescently labeled, single-cell, prototrophic, “ancestor derivative” strains used in fitness competitions (Table 1). yJHK110 was used as the reference and yJHK111 as the control in the RNAseq analysis (Table 2, 3). yJHK111 was the wild-type strain competed against EngClumpy.

4. yJHK085 is also the ancestor of yJHK459. yJHK459 is the common ancestor of all evolved populations and is the strain that was sequenced as the ancestor for the bulk segregant analysis. The *POL3-L523D* mutation was made in yJHK459 (using a plasmid provided by Dmitry Gordenin) to generate two independent transformants, yJHK469 and yJHK470. YFP markers were added to yJHK469 to generate independent transformants yJHK471, 472, and 473, and to yJHK470 to generate independent transformants yJHK475 and 476. *MAL11* and *MAL12* were deleted from yJHK472 to make independent transformants yJHK481 and 482, and from yJHK475 to make yJHK483. These eight independently derived transformants (yJHK471, 472, 473, 475, 476, 481, 482, and 483) were used as starting strains for the experimental evolution (two different colonies from yJHK472 and yJHK481 were used for two cultures each). We used independently derived strains instead of a single strain to start the parallel cultures to avoid biasing the evolution with mutations acquired during construction of the strain. yJHK481, 482, and 483 have *MAL11* and *MAL12* deleted because we wanted to ensure that all populations didn’t evolve to import sucrose as the only strategy.

5. yJHK519 was used as the *MAT****α ***ancestor for bulk segregant analysis. It is derived from yJHK110 with the changes, including a mating-type switch, shown in the table.

6. yCerulean is a yeast optimized version of Cerulean , a CFP variant, and ymCherry is a yeast optimized version of mCherry ; both were provided on a plasmid by N. Ingolia. ymCitrine is a yeast optimized version of mCitrine , a YFP variant.

7. In the yJHK312, *GAL3* was placed on the *ACT1* promoter in order to achieve a graded response from galactose .

8. *amn1-W303* was replaced by *AMN1-RM11* by using plasmid pEF607, which was provided by the Kruglyak Lab .

9. *his3MX6*, *kanMX6* (G418 resistance), and *hphMX4* (hygromycin resistance) come from pFA6a-series plasmids .

10. *ble* (phleomycin resistance) comes from plasmid pUG66 .

11. To produce the form of the *SUC2* gene that could only produce cytoplasmic invertase, we created the *suc2-cyt1* allele by deleting the two ATG codons that precede the transmembrane domain of the secreted form of Suc2. This allele is similar to other alleles that have been shown to only produce cytoplasmic invertase .

12. To request strains or plasmids, please see instructions on the Murray Lab web site (http://www.mcb.harvard.edu/murray/contact.html).

Strain references

Goldstein AL, McCusker JH. 1999. Three new dominant drug resistance cassettes for gene disruption in *Saccharomyces cerevisiae*. *Yeast* **15**: 1541-53. doi:10.1002/(SICI)1097-0061(199910)15:14<1541::AID-YEA476>3.0.CO;2-K

Gueldener U, Heinisch J, Koehler GJ, Voss D, Hegemann JH. 2002. A second set of loxP marker cassettes for Cre-mediated multiple gene knockouts in budding yeast. *Nucleic Acids Research* **30**: e23.

Ingolia NT, Murray AW. 2007. Positive-feedback loops as a flexible biological module. *Curr Biol* **17**: 668-77. doi:10.1016/j.cub.2007.03.016

Kaiser CA, Botstein D. 1986. Secretion-defective mutations in the signal sequence for *Saccharomyces cerevisiae* invertase. *Mol Cell Biol* **6**: 2382-91.

Longtine MS, McKenzie A, Demarini DJ, Shah NG, Wach A, Brachat A, et al. 1998. Additional modules for versatile and economical PCR-based gene deletion and modification in *Saccharomyces cerevisiae*. *Yeast* **14**: 953-61. doi:10.1002/(SICI)1097-0061(199807)14:10<953::AID-YEA293>3.0.CO;2-U

Nguyen AW, Daugherty PS. 2005. Evolutionary optimization of fluorescent proteins for intracellular FRET. *Nat Biotechnol* **23**: 355-60. doi:10.1038/nbt1066

Perlman D, Raney P, Halvorson HO. 1986. Mutations affecting the signal sequence alter synthesis and secretion of yeast invertase. *Proc Natl Acad Sci USA* **83**: 5033-7.

Shaner NC, Campbell RE, Steinbach PA, Giepmans BNG, Palmer AE, Tsien RY. 2004. Improved monomeric red, orange and yellow fluorescent proteins derived from Discosoma sp. red fluorescent protein. *Nat Biotechnol* **22**: 1567-72. doi:10.1038/nbt1037

Sheff MA, Thorn KS. 2004. Optimized cassettes for fluorescent protein tagging in *Saccharomyces cerevisiae*. *Yeast* **21**: 661-70. doi:10.1002/yea.1130

Van Mulders SE, Christianen E, Saerens SMG, Daenen L, Verbelen PJ, Willaert R, et al. 2008. Phenotypic diversity of Flo protein family-mediated adhesion in *Saccharomyces cerevisiae*. *FEMS Yeast Research* **9**: 178-90. doi:10.1111/j.1567-1364.2008.00462.x

Voth WP, Olsen AE, Sbia M, Freedman KH, Stillman DJ. 2005. *ACE2*, *CBK1*, and *BUD4* in budding and cell separation. *Eukaryotic Cell* **4**: 1018-28. doi:10.1128/EC.4.6.1018-1028.2005

Yvert G, Brem RB, Whittle J, Akey JM, Foss E, Smith EN, et al. 2003. Trans-acting regulatory variation in *Saccharomyces cerevisiae* and the role of transcription factors. *Nat Genet* **35**: 57-64. doi:10.1038/ng1222
